# Leveraging experimental vasculature data for high-resolution brain tumor simulations

**DOI:** 10.1016/j.bpj.2026.03.005

**Published:** 2026-03-25

**Authors:** Eric Behle, Julian Herold, Alexander Schug

**Affiliations:** 1 Jülich Supercomputing Centre, Jülich Research Centre, Jülich, North Rhine-Westphalia, Germany; 2 Scientific Computing Center, Karlsruhe Institute of Technology, Eggenstein-Leopoldshafen, Germany

## Abstract

Cancer remains a leading cause of mortality. Multidisciplinary studies probe its complex pathology to increase treatment options. Computational modeling of tumor growth on high-performance computing resources offers microscopic insight into its progress and a valuable avenue for advancing our understanding. However, the effective initialization and parameterization of the underlying models require high-resolution data from real tissue structures. Here, we leveraged high-performance computing resources and a massive data set of a mouse brain’s entire vascular network. We processed these image stacks into detailed three-dimensional representations, identified brain regions of interest, and conducted a series of large-scale simulations to investigate how tumor growth is influenced by local vascular network characteristics. By simulating tumor growth with subcellular resolution, we can probe to which extent vessel density and vessel network length influence tumor growth. We determined that vessel density is more likely to affect the growth rate than the network length. Finally, our results allowed us to extrapolate tumor cell growth predictions for the entire mouse brain, highlighting the critical role of vascular topology in tumor progression. Such increasingly realistic simulations of cancer cells and their microenvironment enable researchers to increasingly bridge the gap between basic biology and clinical practice, ultimately supporting the development of more effective personalized cancer therapies.

## Graphical abstract

**Figure:**
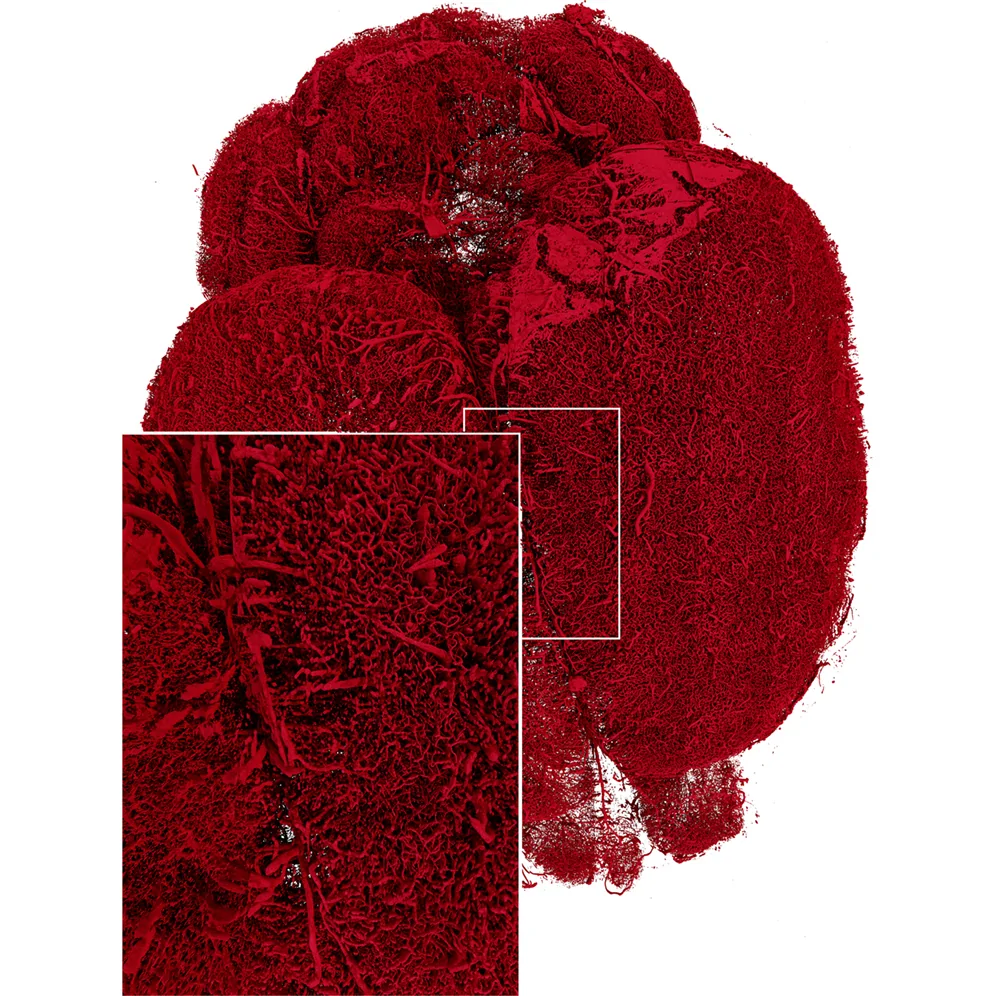

## Significance

Understanding how tumors interact with their surrounding vasculature is key to improving cancer therapies, yet most computational models rely on oversimplified assumptions about nutrient supply. In this study, we combined high-performance computing with a complete capillary-scale map of a mouse brain vasculature to simulate tumor growth at subcellular resolution. Our results reveal that vessel density, rather than vessel network length, is the primary determinant of tumor proliferation. By extending these insights to predict tumor growth across the entire brain, we highlight how realistic vascular topology shapes tumor dynamics. This work demonstrates the power of integrating detailed experimental data with large-scale simulations to bridge the gap between basic biology and clinical applications in cancer research.

## Introduction

Cancer remains one of the leading causes of death worldwide. Thus, it poses an ever-present global health challenge that urgently requires improved therapeutic strategies and deeper insights into its pathology. Despite remarkable advances in cancer treatment, the complexity of tumor growth, its interaction with surrounding tissues, and its eventual resistance to therapies often lead to challenges in effective clinical management. Central to addressing these challenges are advanced simulations of cancer tissue. The massive growth of compute resources allows studies that offer the possibility of highly detailed, subcellular insights into the dynamics driving tumor progression. They also enable researchers to visualize and analyze critical interactions within the tumor microenvironment that are otherwise challenging to study *in vivo*. Such interactions include nutrient gradients, vascular influences, and cellular displacement. By mimicking these intricate biological processes in a controlled computational setting, simulations have emerged as a vital pillar in cancer research. They bridge the gap between empirical experimental data and theoretical models, complementing wet-lab findings and aiding in hypothesis testing. (1,2,3,4). Computational simulations have been instrumental not only in cancer studies but also in fields such as developmental biology, where they provide insights into tissue mechanics and cellular processes in embryogenesis and wound healing (e.g., (5,6,7)). This multidisciplinary approach facilitates the development of comprehensive models that can inform various stages of cancer development and therapeutic response. These can vary in resolution and scope, from modeling cellular and molecular dynamics at a fine scale to examining tissue-wide phenomena (8,9). The ultimate goal of many research initiatives is to develop “digital twins” of tumors: highly realistic, computational representations of tumors within healthy tissue that mimic their real-life counterparts (10,11,12). These digital twins promise to revolutionize personalized medicine, enabling simulations of disease progression and treatment response specific to an individual’s unique biological profile. Building such a model requires an accurate and dynamic morphology of both the healthy and tumorous tissue. It needs to capture the growth behavior of tumors as they interact with the cellular environment, migrate, and displace other cells. A key factor in these simulations is nutrient availability, as nutrient gradients largely dictate tumor growth dynamics and progression patterns (13,14). The transition from avascular (limited to nutrient diffusion) to vascularized growth phases is a significant turning point in early tumor development, where angiogenesis, the formation of new blood vessels, provides the tumor with an essential nutrient supply. Without this transition, tumors cannot grow beyond a critical volume of around 1–3 mm^3^ before nutrient limitations halt further expansion. Accordingly, nutrient supply mechanisms and vascularization processes have become central topics in cancer research, as numerous studies have shown how nutrient gradients shape tumor viability and aggressiveness (15,16,17). We have done a previous study on the interaction of tumors with their nutrient environment, which relied on idealized representations of nutrient distributions, such as simple radial gradients that assume uniformity in nutrient availability (18). However, this model lacked the complexity of real tissue environments, where nutrient distribution is influenced by an intricate vascular network. Other models, which have explicitly modeled blood vessels, have also done so only in a qualitative manner (19,20). A more realistic depiction of the tissue microenvironment requires detailed information about vascular topology, particularly at the capillary level, which is essential for understanding how tumors respond to spatially heterogeneous nutrient supplies. Here, we aim to go beyond idealized models by integrating data that more closely approximate actual tissue environments. Leveraging advances in fluorescent microscopy and image processing, we utilize high-resolution data from Di Giovanna et al. (21), which provide a capillary-scale map of the entire vascular network within a mouse brain. Such data allow us to incorporate highly realistic tissue morphology into our simulations, capturing the nuanced interactions between tumor cells and their vascular surroundings during early development, i.e., before induction of angiogenesis. Our approach takes advantage of computational resources and simulation frameworks. High-performance computing clusters, now more accessible than ever, support these complex models by handling vast data sets and performing simulations at subcellular resolution. A key factor in executing these simulations is the parallelization and scalability of simulation software; the Cells in Silico (CiS) framework (22), used in our study, demonstrates effective performance scaling across thousands of computing nodes, making it a suitable tool for handling the demands of high-resolution, three-dimensional (3D) simulations.

In the following, we first outline the data set, describing the imaging techniques and data processing methods used to achieve a detailed 3D representation of vascular topology. We then discuss the integration of these vascular maps into our simulation pipeline and present our analysis of how variations in vascular network characteristics influence tumor growth patterns. A critical question in tumor growth is the dependence of growth on the local microenvironment and accessibility of nutrients. Here, large-scale simulations provide subcellular insight and identify key factors catalyzing growth. To our best knowledge, the presented simulations of $>O\left(10^{9}\right)$ voxels are currently unprecedented. Finally, we summarize the insights gained from these simulations and propose directions for future research, including potential applications in the development of patient-specific cancer therapies.

## Materials and methods

### Model description

CiS was developed by our group as a framework for simulating the dynamics of cells and tissues at subcellular resolution (18,22,23). It is a hybrid model composed of a cellular Potts model (CPM), nutrient and signal exchange functionalities, and an agent-based layer. With it, we can capture individual cell dynamics in detail. Furthermore, it is an extension of the *NAStJA* framework (24), a highly parallelizable general solver for stencil algorithms. Being specifically designed for deployment in high-performance computing environments, the efficient scaling behavior of CiS allows the simulations of even macroscopic tissues (22,25,26). Hence, CiS has already been used for simulating tissues composing millions of cells (22). Here, we briefly outline the main properties of the individual layers. A detailed description can be found in (22).

#### CPM layer

The CPM is an extension of the Potts model, developed by Graner and Glazier (27). In it, we describe cells as connected voxels of same value on a 3D regular grid. The value of individual voxels may change over time depending on the energy of the system. This energy is described by the following Hamiltonian:

(1) $$\begin{array}{ccc} H_{\text{CPM}} & =\underset{i}{\sum }\lambda_{i}E_{i}\\ & =\lambda_{V}\underset{c\in C}{\sum }{\left(v\left(c\right)-V\left(\tau \left(c\right)\right)\right)}^{2}\; & Cell\;volumes\\ & +\lambda_{S}\underset{c\in C}{\sum }{\left(s\left(c\right)-S\left(\tau \left(c\right)\right)\right)}^{2}\; & Cell\;surfaces\\ & +\underset{\left\langle i,j\right\rangle }{\sum }A_{\tau \left(c_{i}\right),\;\tau \left(c_{j}\right)}\left(1-\delta_{c_{i},c_{j}}\right)\; & Cell-cell\;adhesion, \end{array}$$

where $\lambda_{i}$ is the weight factor for the energy contribution $E_{i}$, c is a cell from the set of all cells C, τ(c) is the type of cell c, s(c) and v(c) are the current surface and volume of cell c, S(τ) and V(τ) are the target surface and volumes of cells of type τ, $\sum_{\left\langle i,j\right\rangle }$ denotes a sum over all unordered neighboring voxel pairs (i,j) on the lattice, A is the adhesion coefficient matrix for all cell types, N(i) are all lattice points neighboring point i, and δ is the Kronecker delta. Equation 1 can be extended to include further effects such as cell motility (28). To propagate the system, we utilize the Metropolis algorithm (29). First, we change the values of randomly picked voxels into those of a respective neighboring voxel and calculate the energy difference ΔE between the old and new system state:

(2) $$\Delta E=H_{\text{CPM,new}}-H_{\text{CPM,old}}.$$

We then decide whether to accept or reject the change. The acceptance probability $p_{\text{accept}}$ is calculated as follows:

(3) $$p_{\text{accept}}=\left\{\begin{array}{cc} 1, & \text{if}\;\Delta E\leq 0.\\ e^{\frac{-\Delta E}{T}}, & \text{otherwise}. \end{array}\right)$$

Here, T is the temperature of the system. Upon successive propagation steps, cells will expand, compress, deform, and move, thereby mimicking their behavior in real tissue. CiS also includes the possibility to add “solid” voxels to the system. Such voxels cannot change their value, and no other voxels can assume a value associated with a solid. This enables us to add structures such as fixed blood vessels.

#### Compound-exchange layer

In addition to detailed cellular structure, CiS contains functionalities for adding compounds such as signals or nutrients to the system. These are exchanged between cells via the cell-cell interface. Solids on the CPM layer can act as sources, thereby mimicking nutrient-supplying blood vessels. The flux $J_{i,j}^{k}$ of compound k between two cells i and j is calculated as:

(4) $$J_{i,j}^{k}=D_{\tau \left(i\right),\tau \left(j\right)}\left(\frac{A_{i,j}}{A_{i}}+\frac{A_{i,j}}{A_{j}}\right)\left({\left[S\right]}_{j}^{k}-{\left[S\right]}_{i}^{k}\right)$$

where $D_{\tau \left(i\right),\tau \left(j\right)}$ is the diffusion constant between cells of type τ(i) and τ(j), $A_{i,j}$ is the interface area between cell i and cell j, $A_{i}$ and $A_{j}$ are the overall surface areas of cell i and cell j, and ${\left[S\right]}_{i}^{k}$ and ${\left[S\right]}_{j}^{k}$ are the compound concentrations within each cell. The aforementioned solids in the CPM layer can also function as compound sources or sinks. The impact of the compound on the system is defined on the agent-based layer.

#### Agent-based layer

On the agent-based layer, each cell within the tissue is tracked as an individual agent. The properties of each agent are adjusted based on information from the other two layers, and their effects are calculated. Furthermore, cellular functions that are not intrinsically captured by the CPM or the diffusion model, such as cell division and cell death, are implemented here. The use of an agent-based layer also allows us to include cell mutation into the framework: upon each cell division, the properties of the two daughter cells can be changed, such that new cell types emerge.

By combining all three layers, we gain a versatile tool, which can then be parameterized to describe a wide range of system dynamics.

### Model parameters

CiS contains many parameters that influence the behavior of the simulated tissue. Here, we highlight those used in this study.

#### Cell-cell adhesion

Adhesion interactions between cells are known to vary within tissues, and especially within tumors. They mediate tissue fluidity and effects such as the epithelial-to-mesenchymal transition demonstrate their importance (30). In our simulations, healthy cells adhere to each other and to tumor cells at a strength of 50 AU, which we define as the baseline adhesion. Tumor cells can have an adhesion strength between 0 and 140 AU (see cell mutation below).

#### Cell motility magnitude and persistence

The CPM Hamiltonian of CiS includes the possibility of adding a cell motility term (28):

(5) $$\begin{array}{cc} H_{\text{CPM,}\;\text{mot}} & =H_{\text{CPM}}+\lambda_{\text{mot}}\cdot \underset{c\in C}{\sum }{\vec{m}}_{c}\cdot {\vec{R}}_{c},\\ {\vec{m}}_{c} & =p\cdot \left({\vec{R}}_{c}\left(t\right)-{\vec{R}}_{c}\left(t-\Delta t\right)\right)+\left(1-p\right)\cdot \vec{\eta } \end{array}$$

Here, ${\vec{m}}_{c}$ represents the potential and direction of a cell c, $R_{\text{c}}\left(t\right)$ and $R_{\text{c}}\left(\Delta t\right)$ represent the center of mass of cell c at Monte Carlo (MC) step t and Δt, respectively, and $\vec{\eta }$ is a random vector obtained by a Wiener process (31). Each cell follows a modified persistent random walk. The energy contribution of each cell is the dot product of $\vec{R_{c}}$ and $\vec{m_{c}}$. $\vec{m_{c}}$ is obtained using the cell’s previous movement and the random vector $\vec{\eta }$. By varying the persistence parameter p∈[0,1], we can mediate the contribution of persistent and random movement. At p=0 a cell performs a purely random walk, while at p=1 it performs a purely persistent walk (32). Finally, we mediate the coupling strength of the motility term to the CPM via $\lambda_{\text{mot}}$. In our simulations, healthy cells have a motility strength of $\lambda_{\text{mot,healthy}}=0$ AU. The motility of tumor cells varies between 0 and 105 AU (see cell mutation below).

#### Nutrient content

Cells require nutrients to proliferate, and a growing tumor is strongly limited by the nutrients supplied by the surrounding vasculature. We therefore defined a “nutrient” compound on the diffusion layer. For simplicity, this single nutrient combines oxygen and all other nutrients supplied by blood vessels. It is released from solids on the CPM layer to adjacent cells. The solids act as a reservoir with a constant concentration of 4 AU. Therefore, the nutrient amount of all cells lies between 0 and 4 AU. The diffusion constant between vessels and cells, and between any two cells is D=0.1 AU, and diffusion takes place once every 10 MC steps. Finally, each cell consumes nutrients: healthy cells use 0.05 AU nutrients every 10 MC steps. We assume that tumor cells are less efficient and therefore use 0.1 AU nutrients every 10 MC steps (33).

#### Cell division rate

Cell agents within CiS are capable of cell division. When this occurs, the cell is split across a randomly oriented division plane on the CPM layer. Half of the voxels belonging to the old cell are assigned a new value that corresponds to the ID of the new cell. The volume, surface, age, and generation properties of the old cell agent are then adjusted, until finally both cells resemble daughter cells of the original one. For division to occur, a division condition must be fulfilled. This condition is customizable and can include a number of different terms, e.g., oxygen content, cell volume, cell generation, and a random component. Since the healthy tissues in our simulations are meant to model a coarse-grained tumor environment, only the tumor cells are capable of division. Our assumption here is that the surrounding tissue is in a steady state of division and death. For a tumor cell to divide, its volume must be at least 60% of its target volume, and its nutrient content must be greater than or equal to 1 AU. Furthermore, we set the division probability such that a cell divides roughly every 10,000 MC steps.

#### Cell mutation

Upon division, a cell can mutate, thereby changing some of its agent properties. We decided to focus on two commonly used properties: the cell motility and adhesion strength (34). A cell has a 10% chance to increase or decrease either its motility or adhesion strength upon division.

#### Cell death

In real tissues, cells can die due to multiple reasons, but for simplicity here we decided to only focus on the nutrient availability. Hence, a cell will die if its nutrients drop below 0.1 AU.

### Loading of blood vessel data into CiS

CiS is parallelized using the message passing interface, and therefore workers have separate memory address spaces (22). Each worker is used to simulate a sub part of the overall CPM volume, which we call a block. We have implemented the loading of the blood vessel data such that each worker loads its individual file at the start of the simulation. These files are generated before running the simulation. We first specify the number and size of blocks in each dimension, and split the blood vessel binary mask into accordingly sized sub volumes. These sub volumes are then written out as files named n.raw, with n being the message passing interface rank to which the sub volume belongs. Each file contains a list of voxel values, which is the flattened version of the respective sub volume. The flattening is done in C-order (row major).

### Data visualization

The visualization of the 3D binary masks and the simulations presented a challenge, since nonspecialized data visualization frameworks have difficulty to do so at this scale (which are up to giga- or even tera-voxel scales). This was achieved using volcanite (to be published) based on previous work (35). Volcanite is a visualization tool for voxel-based data that utilizes efficient compression and on-GPU-decompression algorithms.

## Results and discussion

### Vascular data processing for large-scale simulations

To inform our simulations based on the vascular network topology, we first processed the raw data into analyzable structures. Here, we detail this processing pipeline.

#### Raw microscopy data

**Figure 1:**
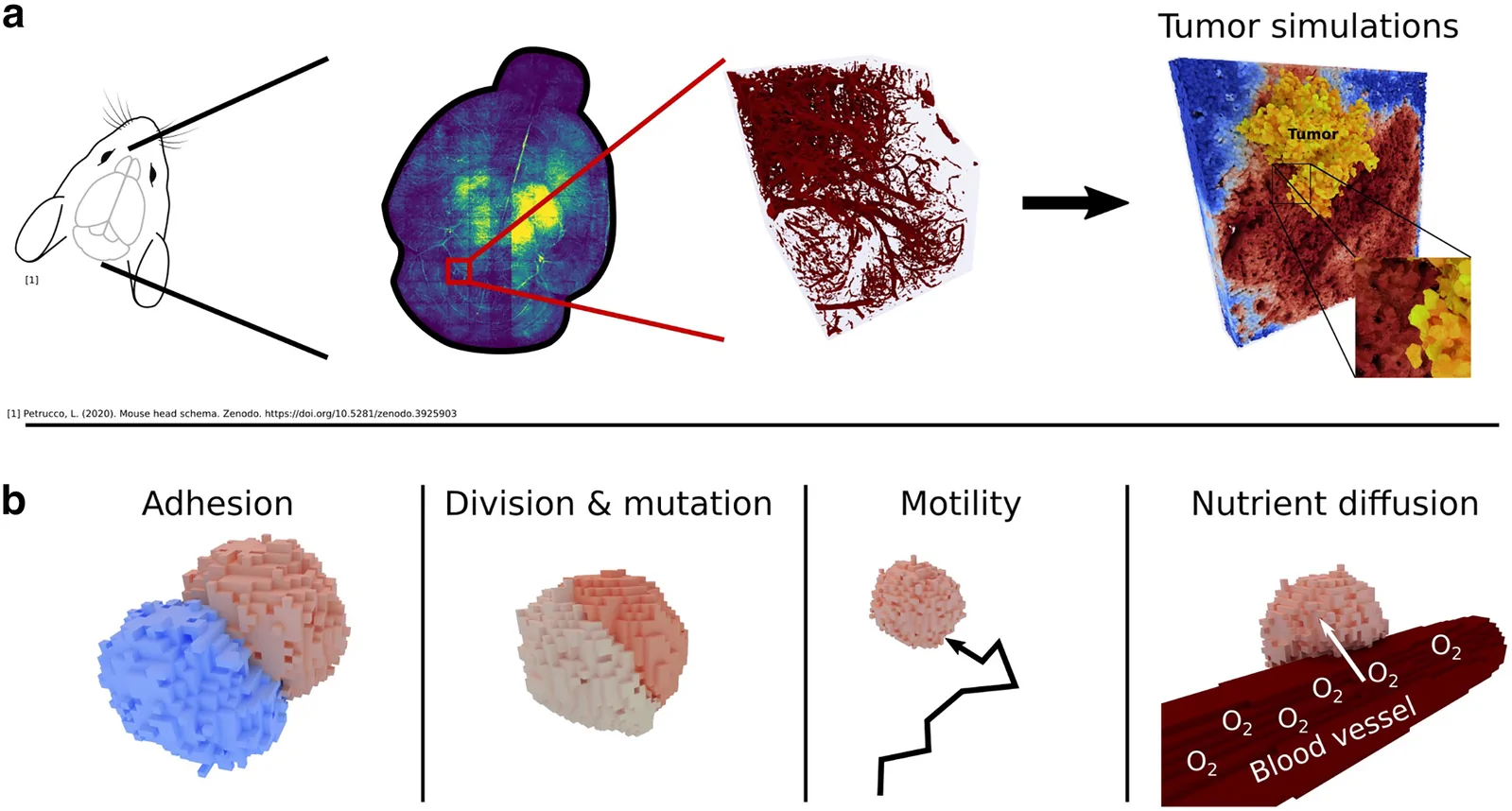
Visual abstract. Mouse brain vasculature microscopy data are a useful resource for the initialization of tumor growth simulations. We utilized data by Di Giovanna et al. (21), who imaged the vasculature network of an entire mouse brain and provided light-sheet microscopy z-stacks. (*a*) In this study, we implemented a pipeline to clean, align, and combine these stacks, obtaining 3D representations. We then performed an analysis of the network topology at different positions and used this analysis to identify areas of interest within the brain. We then performed a large-scale analysis of tumor growth simulations depending on the nutrient environment based on these regions of interest. (*b*) The simulations model a number of different phenomena including adhesion, proliferation, mutation, motility and nutrient diffusion in order to explore the influence of the microenvironment on tumor growth.

**Figure 2:**
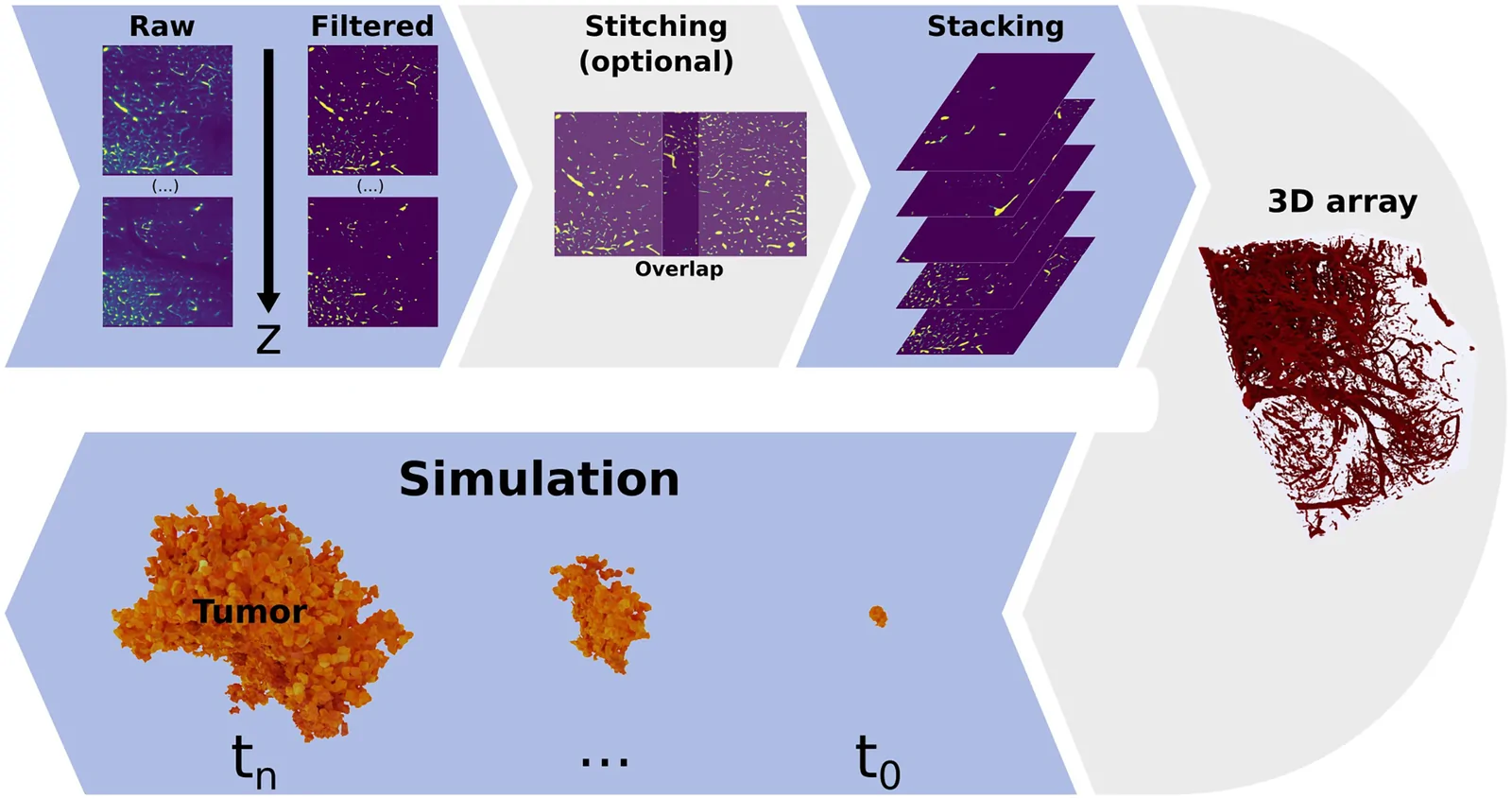
Overview of the mouse brain vasculature data processing pipeline. The pipeline begins with raw microscopy images, which are converted into binary masks using a threshold filter to isolate vascular structures. Due to the size limitation of individual z-stacks (1331×1331×4320 *μ*${\text{m}}^{3}$), a stitching procedure can be applied to extend the simulation volume. By optimizing the alignment of adjacent z-stacks, we minimize artifacts in the merged image. These binary masks are then assembled into a 3D array, which is subsequently imported into the Cells in Silico (CiS) platform, providing spatial and structural information for nutrient source modeling in our simulations.

Di Giovanna et al. measured the entire vascular network of a mouse brain at capillary resolution (21) (see Figs. 1 and 2). The raw data generated by them are available in the form of 144 z-stacks of light-sheet microscopy images (21). Each stack contains 2160 microscopy images with a resolution of 2048×2048 px. One px represents an area of 0.65×0.65 *μ*${\text{m}}^{2}$, and the spacing between each image is 2 *μ*m in the z-direction. Hence, a single stack depicts a volume of 1331×1331×4320 *μ*${\text{m}}^{3}$. Furthermore, adjacent stacks have an overlap of roughly 300 *μ*m in x- and y-directions.

#### Processing into 3D volumes

Processing the data into a usable form presents several challenges. First, since they are raw microscopy images, they contain noise. Secondly, there may be artifacts from the experimental procedure, especially at the edges of each stack. Therefore, the first step in obtaining a 3D representation of all stacks was to denoise them. Toward this goal, we built binary masks from the microscopy images of each of them. This was done by applying a threshold filter on each image (see Table SB2 for threshold values). All images were then combined in a 3D array. The resulting array contains the value 1 in locations where a blood vessel was present, and the value 0 otherwise. Next, we rescaled this 3D binary mask (using transform.rescale from the scikit-image Python module). By doing so we matched the native resolution of our model, such that 1 voxel represented a volume of 1 μ${\text{m}}^{3}$. This was done for each individual microscopy stack. Finally, to obtain a roughly cubic geometry for our simulations, we split each stack into four substacks along the *z* axis. The final volume of the binary mask of each substack was therefore V=1331×1331×1080 *μ*${\text{m}}^{3}$.

#### Stitching multiple stacks

We implemented a method that enables us to stitch neighboring regions into a single stack. This addresses the second challenge of the raw data by minimizing artifacts at the stack edges. See Appendix A in the supporting material for more details.

#### Microscopy stack grouping

**Figure 3:**
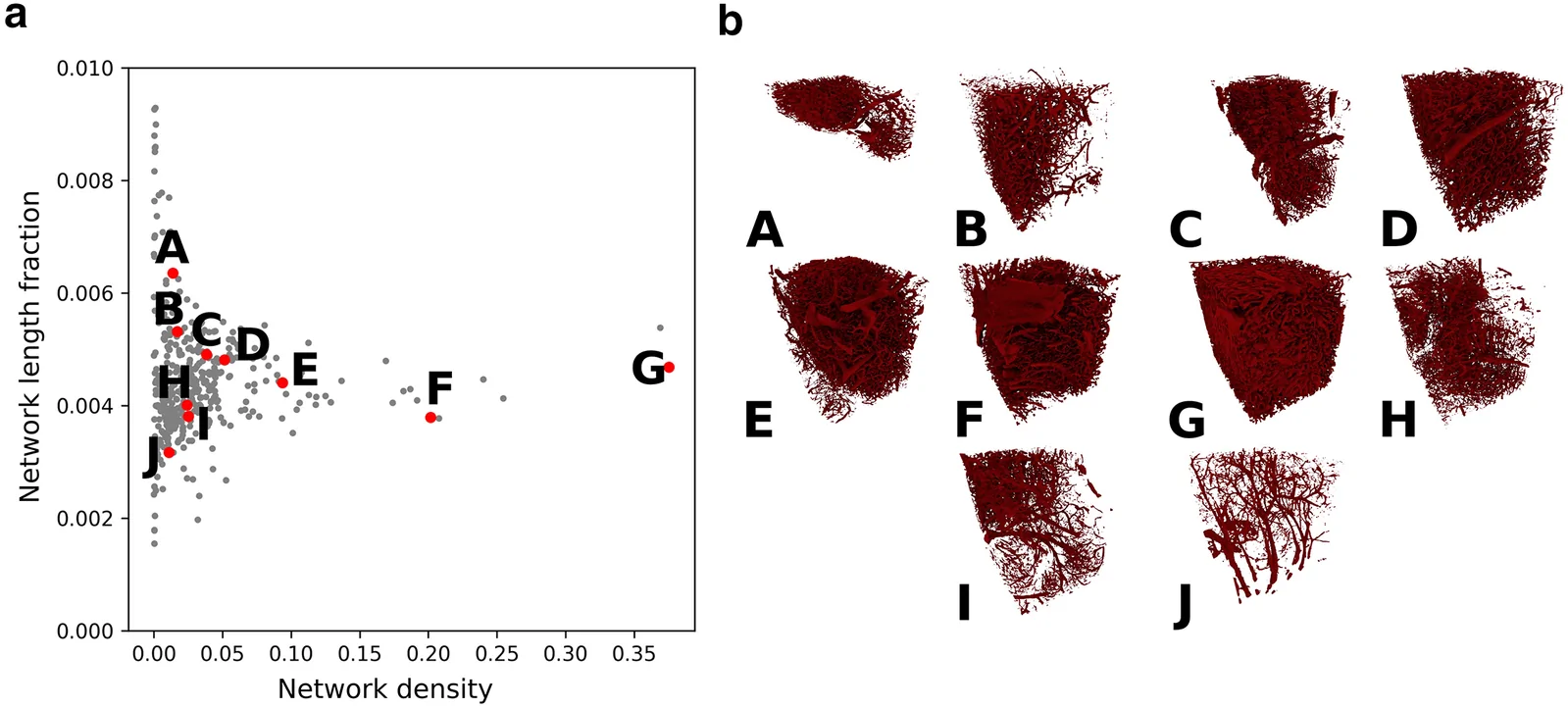
(*a*) Scatter plot of all substacks across the whole mouse brain, characterized by their local blood vessel network density ρ and network length fraction $f_{\text{l}}$. Each gray point corresponds to one substack, while the 10 red points highlight the stacks selected for further analysis. (*b*) Three-dimensional renderings of the 10 selected substacks of interest, corresponding to the red points in (*a*).

Even though supercomputers have grown immensely in computational power in recent years (36,37), simulating the entire 3D representation of the mouse brain at *μ*m-resolution would still be a monumental challenge. On the other hand, it could be argued that, due to the respective similarity between different regions (see also Fig. S5), significant insights can already be gained by focusing on a subset of archetypes. To determine these archetypes, we decided to characterize the substacks and find points of interest. We used two parameters for this characterization: the vessel network density ρ, and a parameter that we call the network length fraction $f_{\text{l}}$. The density ρ was taken as the fraction of vessel voxels within the substack. The network length fraction $f_{\text{l}}$ was calculated through a skeletonizing procedure (38). In such a procedure, the given binary mask is iteratively eroded, reducing the diameter of each vessel to 1 voxel. To remove artifacts distorting the skeletonization, we added erosion, opening, and convolution procedures, thereby removing single voxels. After applying a pipeline of these morphological operations, $f_{\text{l}}$ was then obtained by computing the ratio of voxels in the skeleton to originally present vessel voxels. A value of $f_{\text{l}}$ close to 0 indicates a network dominated by a few thick vessels, resulting in a low overall skeleton length, while a value close to 1 corresponds to networks composed of many thin vessels, which strongly increase the overall skeleton length. Intuitively, ρ and $f_{\text{l}}$ capture complementary aspects of vascular morphology: ρ reflects how much of the volume is occupied by vessels, whereas $f_{\text{l}}$ reflects how finely these vessels are ramified. For example, a high density ρ combined with a high $f_{\text{l}}$ corresponds to a dense mesh of numerous thin vessels; low ρ and low $f_{\text{l}}$ indicate sparse networks with only a few thick vessels. A high ρ but low $f_{\text{l}}$ would arise in regions dominated by large vessels occupying substantial volume but contributing relatively little to total length, while low ρ and high $f_{\text{l}}$ would describe tenuous but highly ramified capillary-like structures. In this way, the two parameters together provide a concise description of both the volume fraction and the structural composition of the vascular network. We used ρ and $f_{\text{l}}$ to inform the selection of 10 substacks (*red points* in Fig. 3 *a*), see supporting material for detailed procedure). A snapshot of each chosen stack’s 3D structure is also shown in Fig. 3 *b*).

### Influence of vessel network topology on tumor growth

After choosing areas of interest within the mouse brain, we investigated the influence of ρ and $f_{\text{l}}$ on tumor growth. Here, we wanted to test two nonexclusive hypotheses regarding nutrient-driven tumor growth:

• Tumor growth is mostly driven by the density of the vascular network (ρ). In this case the local blood vessels drive tumor growth, since higher density leads to more available nutrients.
• Tumor growth is mostly driven by the local topology of the vascular network $\left(f_{\text{l}}\right)$. In this case different local topologies result in differences of the vessel network surface area, influencing the available amount of nutrients.

To evaluate the relationship between ρ, $f_{\text{l}}$, and the final tumor cell count, we performed simulations of tumors seeded in different parts of the brain using the CiS tissue simulation framework (22). See model description for a detailed summary of CiS.

#### Simulations

In each simulation, blood vessels were represented by inserting specialized static voxels into the CPM layer of the CiS framework (see loading of blood vessel data into CiS). These vessel voxels, derived from the binary mask data from processing into 3D volumes, served as nutrient sources. Importantly, we neglected the effect of tumor-induced angiogenesis and vessel remodeling, since we were interested in the early development of the tumor. The remaining volume was populated with healthy tissue cells, maintaining a density consistent with mouse brain cell populations (∼130,000 cells) (39). In our simulations, each CPM cell corresponds to a single biological cell, represented as a contiguous volume of voxels with sidelength of 1μm. The initial cell volumes were chosen to be close the target volume in the Hamiltonian (i.e., 5000 voxels per cell), meaning that we assumed an equilibrated initial configuration. For each selected region, 8 simulations were run with a single initial tumor of fixed volume (typically 10 cells) seeded in a random position. The simulations were run under periodic boundary conditions (pbc) and simulation box size and simulation duration were chosen so that no self interaction could happen, preventing pbc-induced artifacts.

Nutrient dynamics, cell division, mutation, and cell death were modeled using coupled but distinct processes. Nutrient diffusion was implemented through the CiS compound diffusion layer (see model description), using a single diffusing species n as a proxy for oxygen, glucose, and other essential metabolites. Vessel voxels acted as fixed nutrient sources. Cells received an amount of n proportional to the number of adjacent vessel voxels (“surface”), after which nutrients spread through the tissue via cell-cell interfaces according to the CiS diffusion model. To maintain a concise modeling framework and avoid introducing additional parameters that are challenging to properly estimate, the nutrient flux per voxel was taken to be independent of vessel thickness, with nutrient release governed by exposed surface area. Vessel-type-specific fluxes were therefore not included. We also did not explicitly model the blood-brain barrier and assumed it to be fully permeable to nutrient n, which is reasonable because the compounds represented by n (e.g., oxygen, glucose) can cross the blood-brain barrier (40).

Cell division and mutation occurred when a tumor cell’s nutrient level exceeded a division threshold. At each division event, the daughter cell could mutate with a fixed probability per timestep. Mutations affected either cell-cell adhesion or motility, each parameter changing by a single discrete step within the allowed range (see supporting material for parameter definitions). The phenotypic ranges and mutation step sizes are detailed in the supporting material.

Cell death was linked to nutrient starvation: when a cell’s nutrient level fell below a critical threshold, it underwent apoptosis. Following the standard CPM implementation, death was realized by gradually decreasing the cell’s target volume to zero, causing it to shrink and be removed from the lattice. To avoid immediate cell death at the beginning of each simulation, all cells were initialized with a baseline nutrient reserve corresponding to the steady-state value expected in an equilibrated, nonconsuming system.

It is important to note that the traditional CPM, as used here, does not fully capture the intricate morphologies of neurons and glial cells. Nonetheless, CPMs are widely applied in similar studies for tissue modeling (41,42). We argue that this simplification is justified, as the primary cell-cell interactions driving these simulations—adhesion forces and nutrient diffusion—are closely related to the shared surface area between cells. Introducing more complex cell shapes would mainly result in two effects: 1) increasing shared surface area for cells already in contact, effectively modifying adhesion and diffusion parameters for all cells uniformly and 2) creating small shared surfaces between cells previously not in contact. Since the first effect would scale uniformly and the second effect introduces minimal interaction, the impact of these adjustments would be limited. Moreover, adding complex shapes would significantly increase both model and computational complexity, with only marginal gains in accuracy.

**Figure 4:**
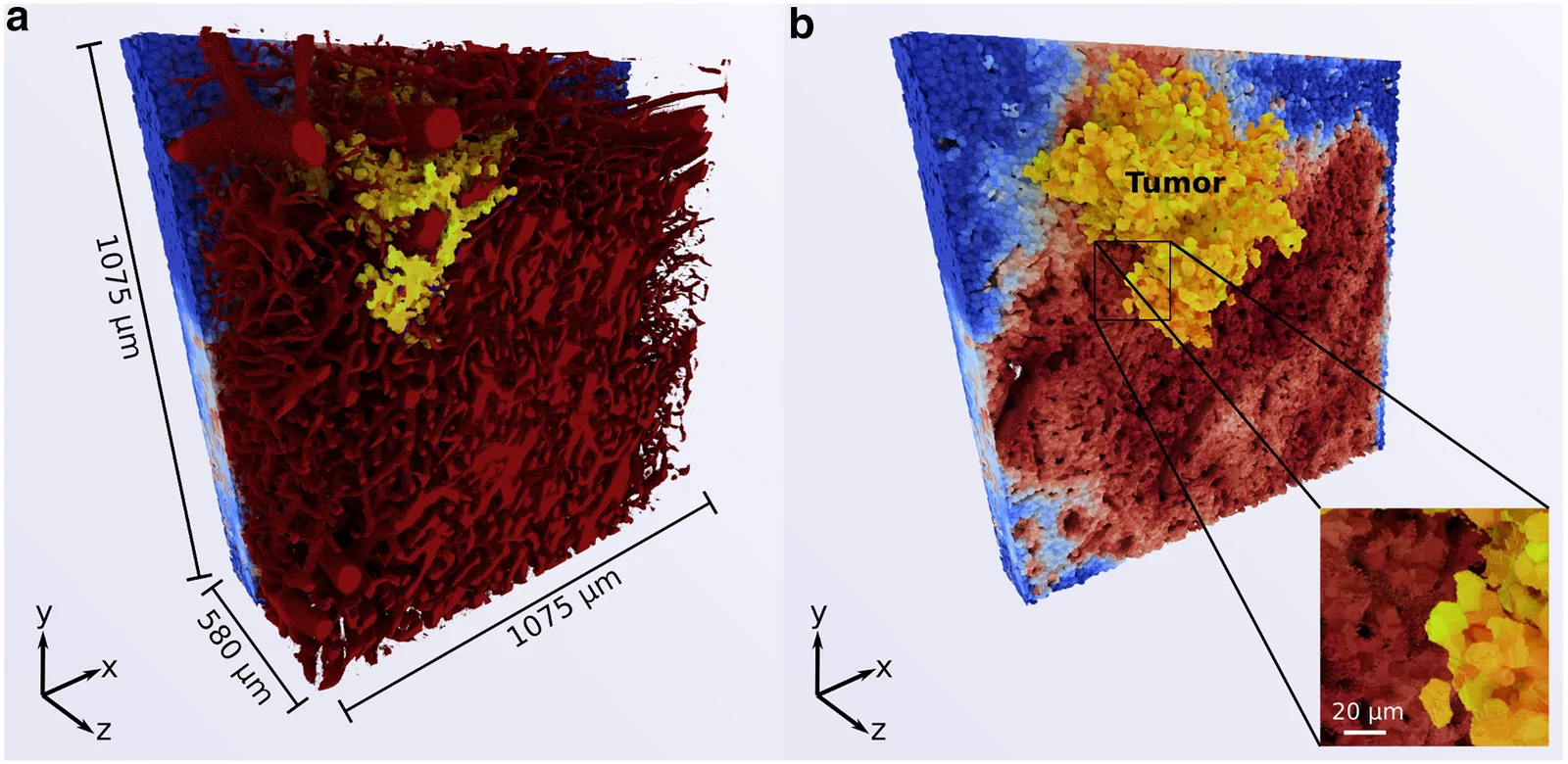
Visualization of a simulated tumor growing within a mouse brain vasculature environment (cluster F in Fig. 3*b*) after 100,000 Monte Carlo (MC) steps. (*a*) The dark red structure represents the blood vessel network, providing nutrient sources. (*b*) The tumor cells (*yellow*) are shown expanding in three dimensions, with the surrounding healthy cells color coded according to their nutrient content (*blue* to *red* gradient), indicating the spatial distribution of nutrient availability. The displayed volume is a subset of the entire simulation space (1344×1344×1080*μ*${\text{m}}^{3}$) to focus on the tumor’s immediate environment.

More details on the model parameters are provided in model parameters and in Table SB1. We performed eight simulations for each centroid substack obtained in microscopy stack grouping. In each simulation, the tumor was seeded at one of eight points. These points were at one-third and two-thirds of each simulation box diagonal. Each simulation ran for an total amount of 250,000 MC steps. A snapshot of a single simulation after 100,000 MC steps is provided in Fig. 4. To preserve clarity and avoid compounding uncertainties from additional approximations, we chose not to convert MC steps into real-time units. Our primary aim is to explore the qualitative influence of the vascular network on tumor progression.

#### Tumor growth behavior

**Figure 5:**
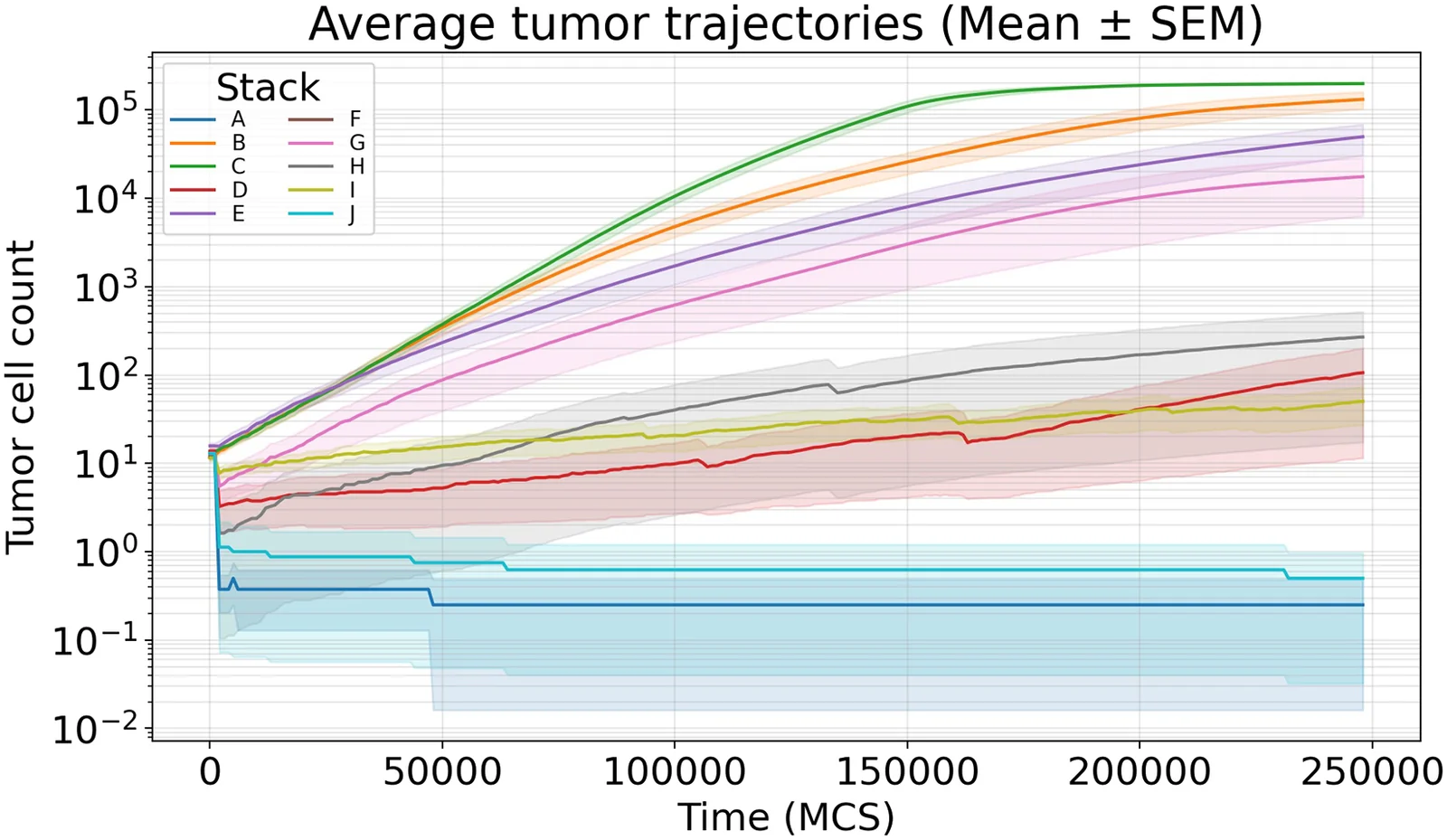
Average tumor cell trajectories across vascular environments. Shown are the mean tumor cell counts (± standard error of the mean) over simulation time for tumors seeded in 10 representative vascular network regions (A–J). Each curve represents the average of eight independent simulations initialized with identical tumor volumes but differing positions. The trajectories separate into three distinct growth behaviors: rapid exponential expansion (clusters C, B, H, and G), gradual sustained growth (clusters D, H, and I), and stagnation or decline (clusters A and J). These patterns illustrate how local vascular properties can fundamentally alter tumor progression.

To first assess tumor growth dynamics across different vascular environments, we analyzed the average tumor trajectories over time (Fig. 5). The trajectories reveal three distinct qualitative classes of tumor behavior: 1) fast-growing tumors with near-exponential expansion (clusters C, B, H, and G), 2) slow-growing tumors with gradual but sustained increase (clusters D, H, and I), and 3) nongrowing or declining tumors, where cell counts stagnate or decrease (clusters A and J). These results highlight the strong influence of local vascular architecture on tumor progression and provide a basis for the subsequent quantitative analysis of vascular properties.

Interestingly, only one vascular environment (stack C) exhibited clear saturation of tumor size during the course of the simulation. Visual inspection confirmed that this plateau is not an artifact of tumors interacting with their own periodic images (pbc), as the tumor remained well within the simulation box boundaries. Instead, this behavior likely reflects mechanical inhibition of further growth due to tissue pressure, suggesting that local mechanical constraints can act as an additional regulator of tumor expansion in certain vascular contexts.

To compare all simulations, we first calculated the local blood vessel properties in the vicinity of each tumor. For this we chose a sphere of radius 250μm around the initial tumor center. We then calculated the local density $\rho_{\text{local}}$ and the local network length fraction $f_{\text{l,local}}$ within this sphere. Finally, we determined the number of tumor cells at the end of each simulation and compared this final tumor size against the local vascular properties, i.e., $\rho_{\text{local}}$ and $f_{\text{l,local}}$ (see Fig. 8). We also considered additional properties such as the maximal tumor growth rate (see Appendix A and Fig. S2), but these did not provide further insights, as growth rate and final tumor size were strongly correlated. While the relationship between the network length fraction and the final tumor cell count is less clear, the dependence of the final tumor cell count on the local blood vessel density shown in Fig. 8 *b* roughly follows a logistic curve:

(6) $$N\left(\rho \right)=A\;\cdot \;\frac{e^{B\cdot \rho }}{e^{B\cdot \rho }+C},$$

where A controls the maximum cell count, B controls the steepness of the slope, and C controls the location of the logistic curve’s inflection point. These data show that the simple assumption of a linear dependence between vessel density and tumor growth rate does not hold. Instead, there exists a threshold above which the tumor grows rapidly. Furthermore, the higher blood vessel surface area resulting from the high network length fractions does not appear to be a main driver for tumor growth. We note, however, that Fig. 8 can be misleading regarding the relationship between tumor cell count and network length, especially in the window of 0.003 and 0.005 of local network length fraction. This can be explained by considering that network length fraction is correlated with vessel density at high densities (see Fig. S3), thus obscuring potential independent contributions of network length to tumor growth.

#### Tumor composition analysis: Neutral expectation and divergence timecourses

**Figure 6:**
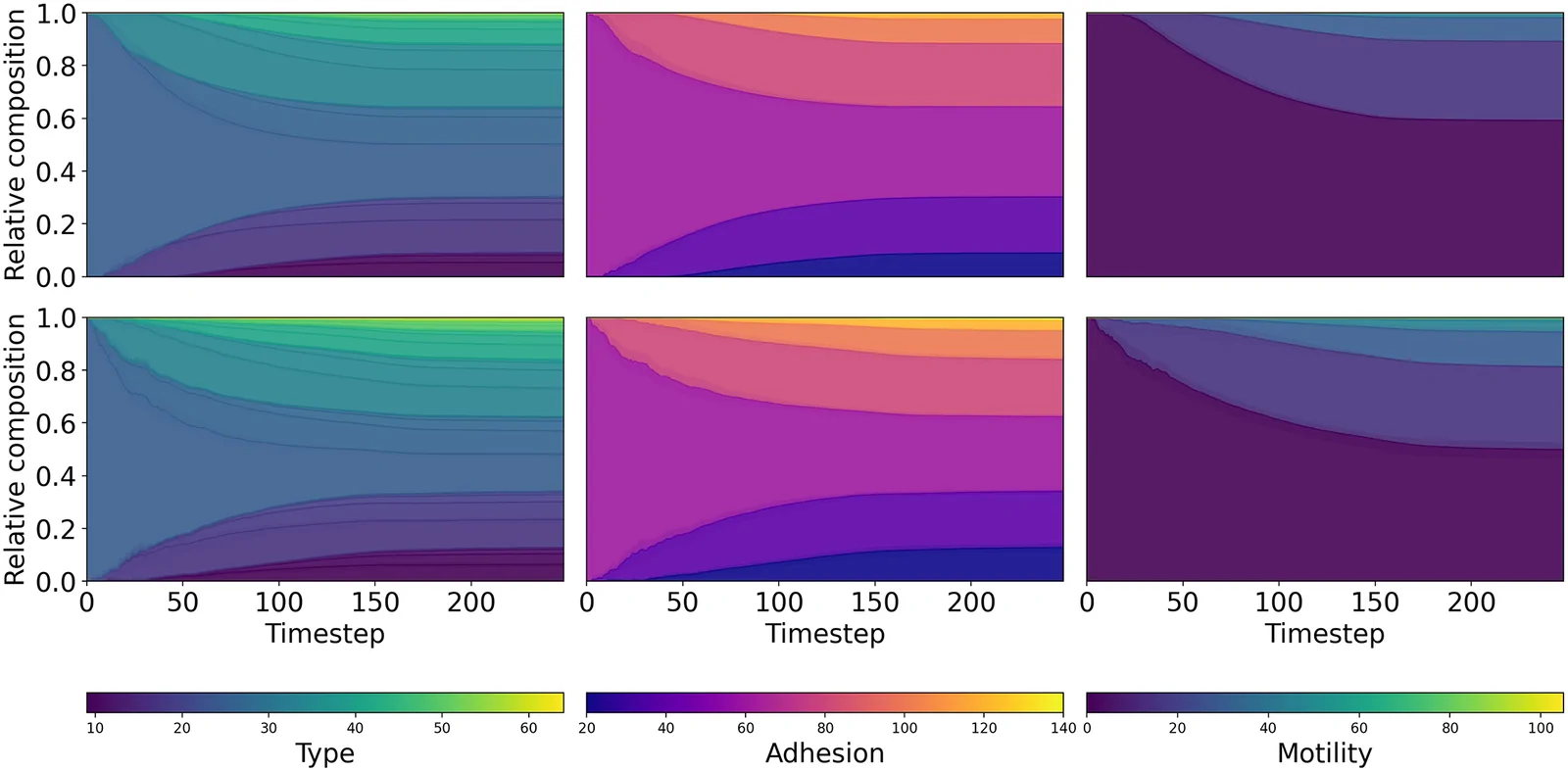
Tumor composition dynamics in stack C compared with neutral expectation. Shown are the relative compositions of phenotypic types, adhesion groups, and motility groups over time. The top row shows results from dedicated neutral-control simulations, in which all phenotypes were assigned equal fitness and the system evolved only through mutation and demographic drift. These runs use the same mutation process and overall tumor growth trajectory as the observed simulations but do not model the actual tissue dynamics and thus exclude any selective differences between types, providing an explicit neutral baseline. The bottom row shows the corresponding observed simulations, where phenotypes reproduce with their true fitness differences and are therefore subject to selection. Deviations between the two rows indicate compositional changes that cannot be explained by mutation and drift alone. In the observed runs, there is a clear selection pressure toward higher motility types and a slight pressure away from high-adhesion types. This suggests that tumor cells with greater motility can more effectively infiltrate surrounding tissue and utilize available resources. Stack C is shown here as an illustrative example; results for other stacks are qualitatively similar and provided in Appendix A in the supporting material, together with a more detailed explanation of the neutral model.

**Figure 7:**
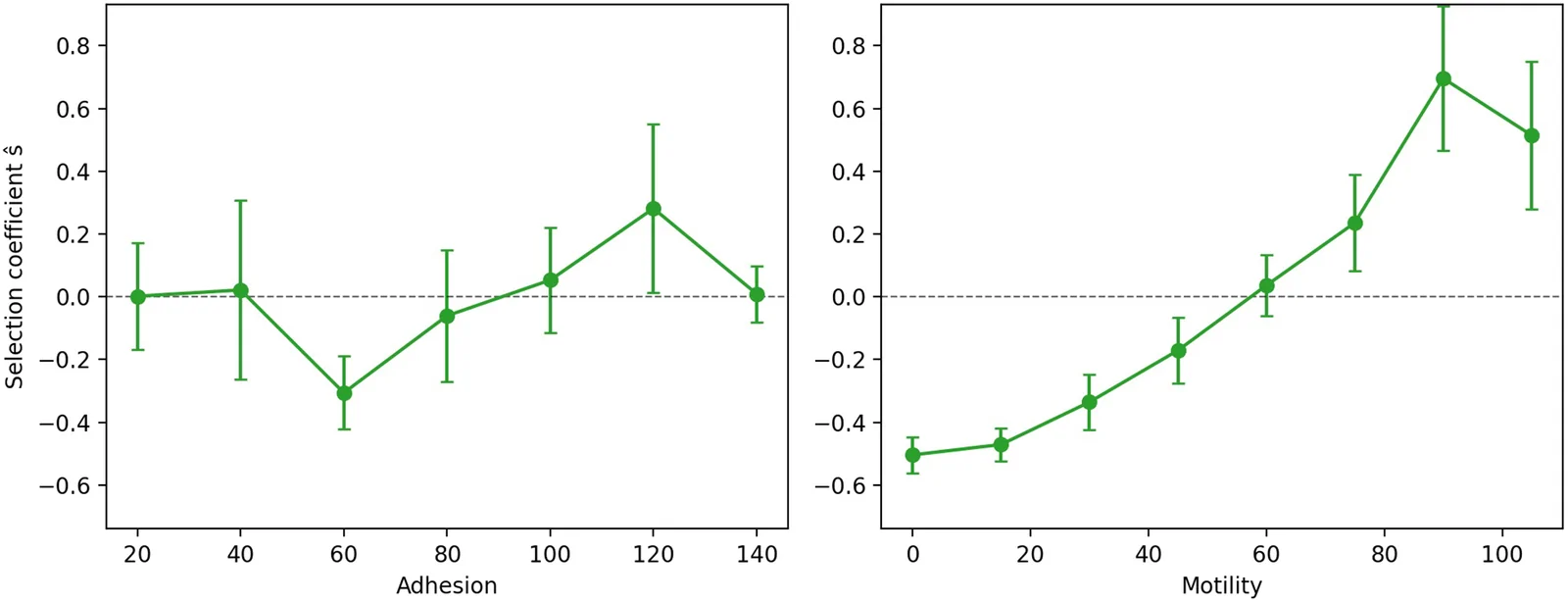
Estimated selection coefficients aggregated by adhesion (*left*) and motility (*right*) levels for a representative imaging stack. Points indicate mean coefficients ${\bar{s}}_{a}$ or ${\bar{s}}_{m}$ with error bars showing the standard error across constituent types. The dashed horizontal line denotes neutrality (s=0). Adhesion shows weak and variable selection, while motility exhibits a clear gradient from negative selection at low motility to positive selection at high motility.

We next asked how the evolving tumor cell composition deviates from a neutral expectation, where all phenotypes have equal fitness and composition changes arise solely from mutation and demographic drift. In our simulations, each tumor cell belongs to one of 56 phenotypic types, defined by discrete adhesion and motility levels. To assess whether selection alters these compositions, we compared the observed type distributions against matched “no-selection” simulations that include only mutation and drift. As a neutral baseline, we implemented a simple model that couples the empirically observed tumor growth dynamics with the same mutation process without modeling the actual tissue dynamics, ensuring that any compositional deviations reflect selective differences rather than overall expansion rates. To create these neutral simulations, we took the total cell count over time (N(t)) of each full simulation and computed the expected tumor composition when following these growth curves and the specified mutation matrices. This results in a neutral simulation for each full simulation, ignoring the spatial distribution of the cells and any selective pressure. As an illustrative example, Fig. 6 shows the composition dynamics of stack C compared with its neutral counterpart. While the neutral simulations display only mutation-drift dynamics, the observed simulations exhibit clear selection pressure: tumor populations shift toward higher motility types and, less pronounced, toward higher adhesive types (cf. Fig. 7). This suggests that cells capable of moving more easily through the surrounding tissue are better able to exploit available resources. Other stacks display qualitatively similar trends, and the full set of composition plots is provided in Appendix A.

For each imaging stack, we estimated type-specific selection coefficients $s_{i}$ by maximum-likelihood inference from net growth events, using a mutation kernel M that encodes allowed phenotypic transitions. Neutrality corresponds to $s_{i}=0$ for all types; deviations indicate differential reproductive success. We formally tested neutrality via a likelihood-ratio test comparing the fitted model to the neutral baseline.

To aid interpretation, we further aggregated selection estimates across phenotypic axes. For each stack, we computed mean selection coefficients for adhesion and motility levels, and visualized them with standard errors across constituent types. A zero-reference line (s=0) marks neutrality: systematic departures along either axis reveal selective advantages or disadvantages. Detailed definitions of the birth-mutation framework, likelihood construction, and aggregation procedure are provided in the supporting material (cf. Appendix D).

#### Predicted tumor growth in the entire mouse brain

**Figure 8:**
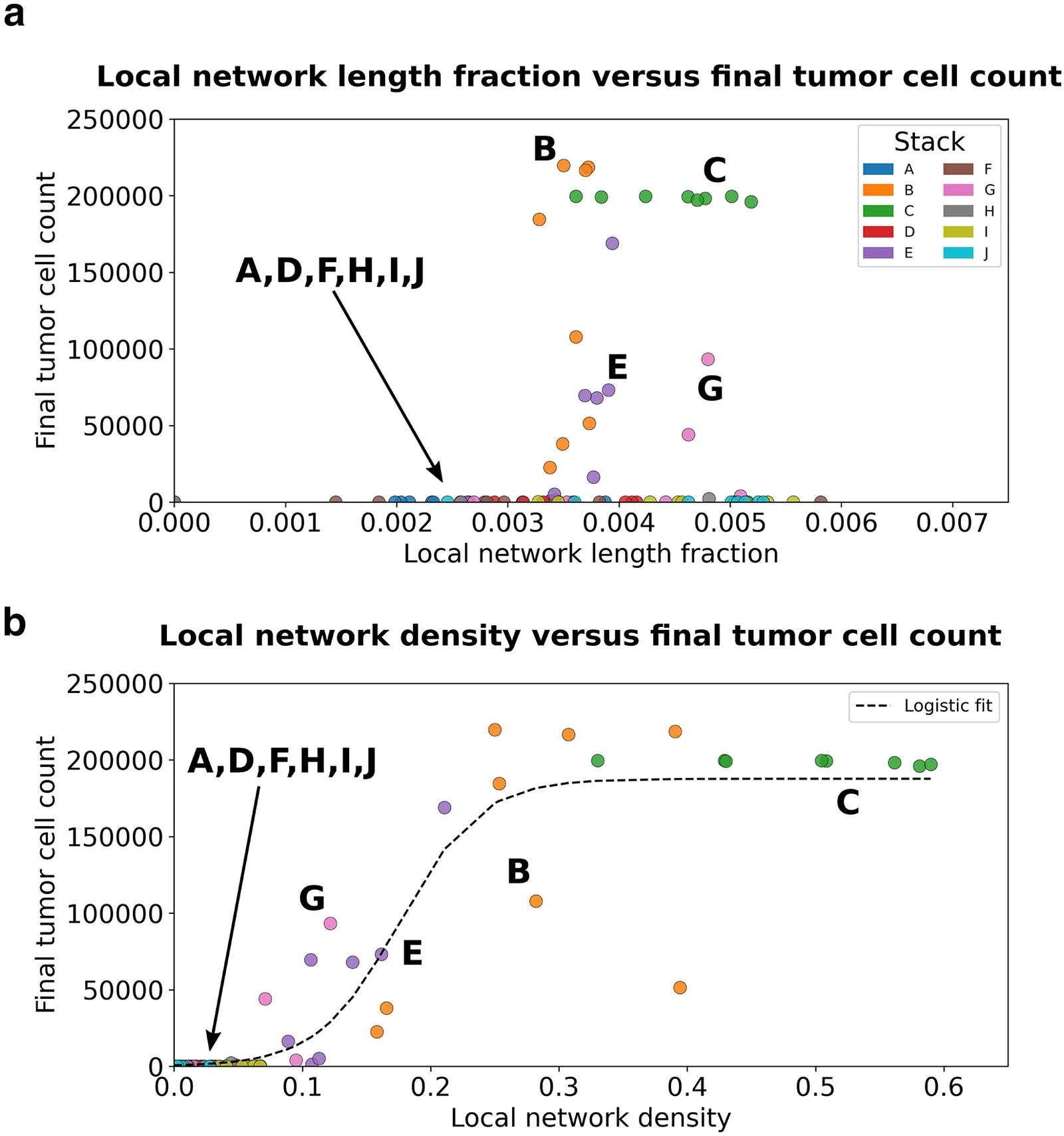
Mouse brain vasculature property comparison. (*a*) Network length fraction versus final tumor cell count. Shown are the final tumor cell counts for eight simulations of each respective microscopy stack chosen in microscopy stack grouping, using the same color coding as in Fig. 5. In each simulation, the tumor was placed at a different starting position, and hence the local network length fraction differs. The network length fraction serves as a proxy for the vessel network length. (*b*) Local blood vessel density versus final tumor cell count. Shown are the color-coded final tumor cell count for eight simulations of each respective microscopy stack chosen in microscopy stack grouping. In each simulation, the tumor was placed at a different starting position, and hence the local blood vessel density differs.

**Figure 9:**
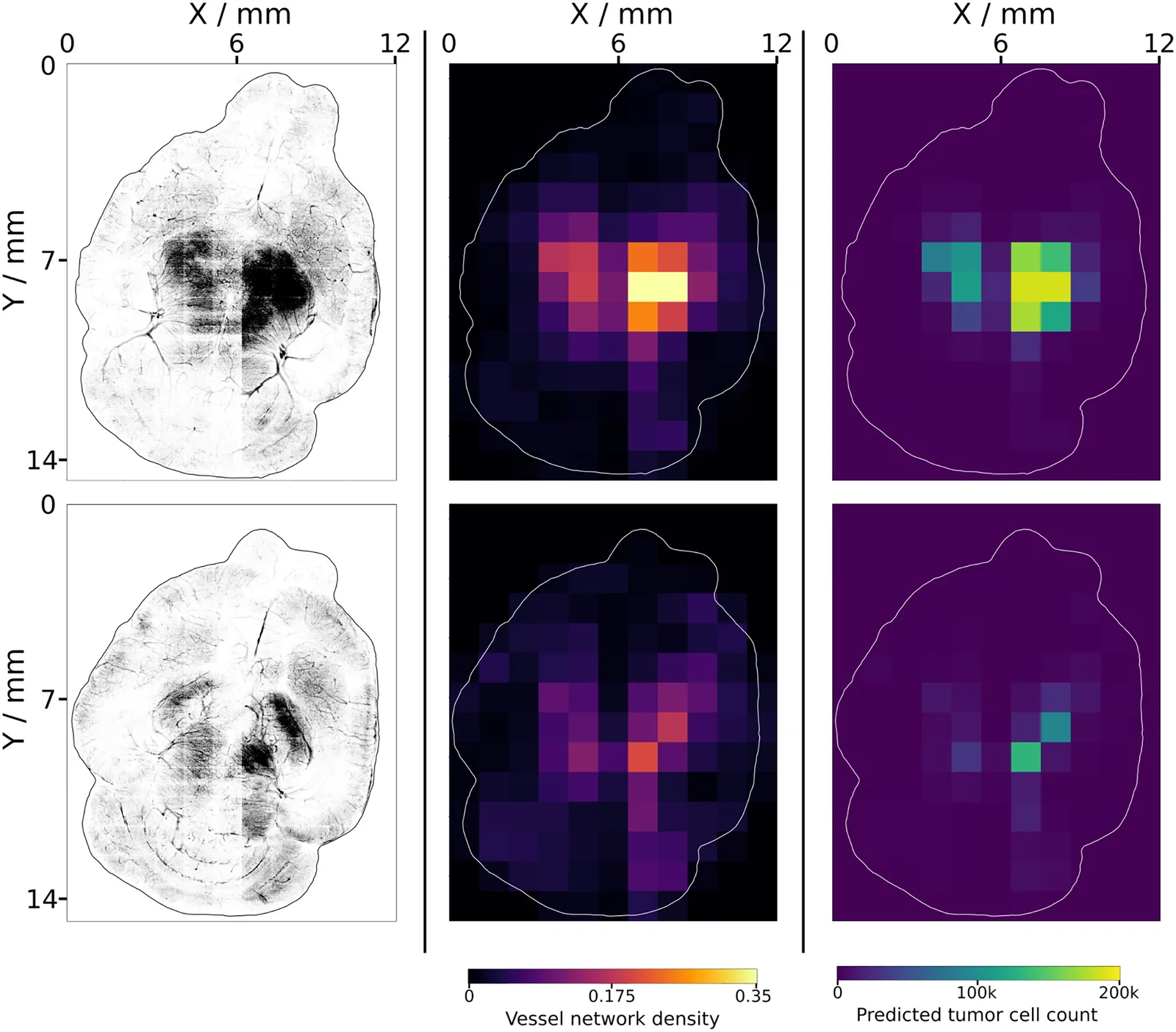
Mouse brain blood vessel density and predicted tumor growth. Left: top-down views of the processed and stitched microscopy stacks for the lowest two layers of the brain. Middle: heatmaps showing the blood vessel network density for each microscopy stack. Right: heatmaps showing the predicted tumor cell count for each microscopy stack. Predicted values were obtained using Eq. 6 with the values from fitting to the simulation data (see predicted tumor growth in the entire mouse brain and Table SB2).

We can use the relation between local blood vessel density and final tumor cell count to estimate the tumor size for the entire mouse brain by fitting Eq. 6 to the data shown in Fig. 8 *b*) (see also Table SB2). We used the resulting values to calculate the expected cell count for the given vessel density ρ of each substack. Within our simulation environment tumors are expected to grow largest in the densest regions in the center of the brain, and to not grow well in the periphery (see Figs. 9 and S3).

## Conclusions

Computational studies of tumor growth can profit immensely from existing experimental data. In this study, we show how we utilized highly detailed microscopy data on mouse brain vasculature to inform our simulation framework CiS. After developing a data processing pipeline for the microscopy stacks found in the raw data, we characterized each processed stack by its vessel network topology. We utilized two parameters for this characterization: the blood vessel density ρ and the network length fraction $f_{\text{l}}$. After choosing 10 substacks of interest based on the 2 parameters, we performed simulations of tumor growth in this wide range of nutrient environments. Placing the initial tumor in different locations yielded different local starting topologies, and we observed different growth behavior based on this. We saw that the local vessel density is more likely to drive tumor growth, and that the final cell count roughly follows a logistic curve. While there are some outliers in Fig. 8 *b*, these show that the final tumor cell count is not determined solely by the starting position of the tumor, but also its surrounding further from it (see also Fig. S4).

The network length fraction, which approximates the distribution of vessel thicknesses, does not appear to have significant influence on the final tumor size. This was surprising to us, since we expected that a higher network length fraction would correspond to a larger surface area of the vessels, leading to increased nutrient diffusion. As this is a common theme of real vessel networks, which branch out into vessels of progressively smaller diameter and larger surface area, this needs to be investigated in more detail. Here, it would be interesting to compare the behavior of our simulations to existing data on tumor growth in brain regions of different vascular structure. Unfortunately we have so far been unable to find a suitable data set, or a comparable simulation study of blood vessel networks at this scale.

There are other metrics used to characterize blood vessel networks, e.g., the number of branches. Extracting these from the given data is nontrivial, however, which is why we have so-far remained with ρ and $f_{\text{l}}$. Future studies could address more features and their relationship to the tumor growth rate.

Using a logistic curve fitted to the local density data, we predicted the tumor growth in the entire brain. Strong tumor growth was predicted mainly in the dense regions in the center of the lower two planes of the brain. One major caveat to mention here is that we used the global vessel density per stack for the prediction, and did not look at more localized vessel properties. This effectively represents an averaging of larger volumes, thus losing information about inhomogeneities. Hence, we might miss some regions that locally surpass the established threshold. Furthermore, as seen in the left panel of Fig. 9, there are some residual brightness artifacts from the microscopy, which may influence the results.

Beyond growth dynamics, our analyses also revealed how tumor cell composition shifts relative to neutral expectations. By comparing simulations with and without selection, we found that phenotypic distributions consistently diverge from neutrality: populations tend to favor higher motility types, while strongly adhesive types are slightly disfavored. Likelihood-based inference confirmed that these deviations are statistically significant, with motility exhibiting a systematic gradient of positive selection at high levels. This indicates that, within the vascular environments studied here, cells with greater ability to migrate through tissue gain a measurable reproductive advantage. Such findings complement our growth predictions by highlighting how microenvironmental structure not only influences tumor expansion but also shapes the evolutionary trajectories of cellular phenotypes.

This study shows that there still is a large amount of information to be gained from the existing experimental data on tissue structure. We aim to utilize these data for further studies of more realistic tumors. In particular, the stitching procedure that we mentioned in vascular data processing for large-scale simulations could be used to simulate the growth of tumors of multiple ${\text{mm}}^{3}$ in volume. Such system sizes would enable us to perform in silico radio- and chemotherapy treatment studies in the future. Furthermore, it is a step on the way to simulating fully vascularized macroscopic tumor growth with our model. A crucial aspect of this is to move from a static vessel network to a dynamic formulation, in which the network can be changed, e.g., via tumor-induced angiogenesis. Hence, our future work will focus on implementing such a formulation.

Eventually we will want to simulate tissues with different blood vessel structures than those predefined by the given data. To do so while still retaining a realistic network we aim to use the data to train an algorithm that places a custom vessel network into a given volume.

## Data and code availability

• The code of CiS can be found at https://gitlab.com/nastja/nastja.
• The data and analysis scripts required for reproducing our analysis can be found at https://gitlab.jsc.fz-juelich.de/behle2/mousebrainvasculaturepaper.
• Due to the size of the entire simulation data (roughly 1.1 TB), we only include the last output file of each simulation within this repository.
• The full data are available upon reasonable request.

## Acknowledgments

The authors gratefully acknowledge the 10.13039/501100022273Gauss Centre for Supercomputing e.V. (www.gauss-centre.eu) for funding this project by providing computing time through the John von Neumann Institute for Computing (NIC) on the GCS Supercomputer JUWELS at 10.13039/501100023739Jülich Supercomputing Centre (JSC). We thank Max Piochowiak for insightful discussions. We are grateful for funding by the 10.13039/501100009318Helmholtz Association in the programs Helmholtz AI (to A.S. and J.H.) and HIDSS4Health (to A.S. and J.K.). The funders had no role in study design, data collection and analysis, decision to publish, or preparation of the manuscript.

## Author contributions

All authors designed the research. Data processing and curation, E.B.; design and performance of simulations, E.B.; programming and software development, E.B. and J.H.; analysis and visualization of data, E.B. and J.H.; writing of the manuscript, A.S., E.B., and J.H.

## Declaration of interests

The authors declare no competing interests.

## Supporting material

Document S1. Figures S1–S8, Tables SB1 and SB2, and Appendices A–E

Document S2. Article plus supporting material
