# Supplementary material for: Leveraging experimental vasculature data for high-resolution brain tumor simulations
Source: Biophys J. 2026 Mar 25;125(7):1701–12. doi: 10.1016/j.bpj.2026.03.005 (PMC13351720; doi:10.1016/j.bpj.2026.03.005)
Supplement: Document S1. Figures S1–S8, Tables SB1 and SB2, and Appendices A–E [file mmc1.pdf]

Biophysical Journal, Volume *125*

Supplemental information

Leveraging experimental vasculature data for high-resolution brain tumor simulations

Eric Behle, Julian Herold, and Alexander Schug

Leveraging Experimental Vasculature Data for High Resolution Brain Tumor Simulations

Eric Behle[1†], Julian Herold[2†], Alexander Schug[1,3*]

[1]Jülich Supercomputing Centre, Jülich Research Centre, Wilhelm-Johnen Str. 1, Jülich, 52425, North-Rhine Westphalia, Germany.
[2]Scientific Computing Center, Karlsruhe Institute of Technology, Hermann-von-Helmholtz-Platz 1, Eggenstein-Leopoldshafen, 76344, Baden-Württemberg, Germany.
[3]Present address: Scientific Computing Center, Karlsruhe Institute of Technology, Hermann-von-Helmholtz-Platz 1, 76344 Eggenstein-Leopoldshafen, Baden-Württemberg, Germany.

*Corresponding author(s). E-mail(s): alexander.schug@kit.edu;
†These authors contributed equally to this work.

Supplementary information.

Appendix A Mouse brain data processing: stitching multiple stacks

In order to fully utilize the data and not be limited by the geometry of a single stack, we implemented a stitching procedure. This procedure combines parts of multiple microscopy stacks into a composite volume. For this, first the desired volume and location within the raw data is defined. Next, all microscopy stacks that have at least partial overlap with the specified volume are determined. Then, the overlapping part of each binary mask is extracted and added to the stitched volume. While doing this, care must be taken to include the overlap of 300 µm between adjacent stacks specified by Di Giovanna et al. However, even doing so is not sufficient: we still noticed some residual inconsistency at the interface between stacks (see Figure S1). To alleviate this, we varied the overlaps in x- and y-direction within specified bounds and calculated the agreement a between the overlapping parts of both masks for each variation. a was calculated by applying an XNOR between each voxel of the overlap, summing the

results and dividing by the number of voxels. Finally, we chose the variation with the highest value of a. While there are remaining artefacts inherent to the measurement procedure in Figure S1, this has led to some improvement.

Appendix B Supplementary tables

Table B1 Simulation parameters.

Parameter	Value
Number of different vessel structures	10
Number of simulations per vessel structure	8
Cell target volume	5000
Cell target surface	1856
Cell-cell adhesion	20, 140
Cell motility magnitude	0-105
Cell-vessel adhesion	20-140
Random walk persistence	0.2
Vessel nutrient content	4
Cell division probability per MC step	0.0001
Cell division minimum nutrients	1.0
Cell death nutrient threshold	0.1

Listed are either fixed parameters or ranges, if the respective parameter was varied, e.g. through cell mutation.

Table B2 Mouse brain data analysis parameters.

Parameter	Value
Number of microscopy image z-stacks	154
Number of microscopy images per z-stack	2160
Microscopy image resolution	2048 x 2048 px
Microscopy image area per pixel	0.65 x 0.65µm^2
Binary mask building threshold filter value	175
Stitching procedure maximum alignment shift	50
Stitching procedure stepsize	2
Fit values for main manuscript equation 1	$A \approx 1.877 \cdot 10^5, B \approx 31.77, C \approx 262.9$

Listed are the parameters related to the mouse brain data processing pipeline. Raw data generated by Di Giovanna et al. [1].

Appendix C Supplementary figures

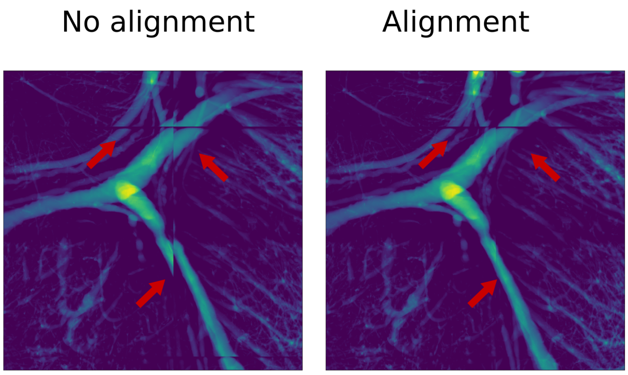

Fig. S1 SI: Result of custom alignment in microscopy stack stitching procedure. Left: no additional alignment, only the overlap of 300 μm specified in [1] is included. This results in imperfect alignment of some blood vessels (see red arrows). Right: custom alignment included. Adjacent vessels match up much better.

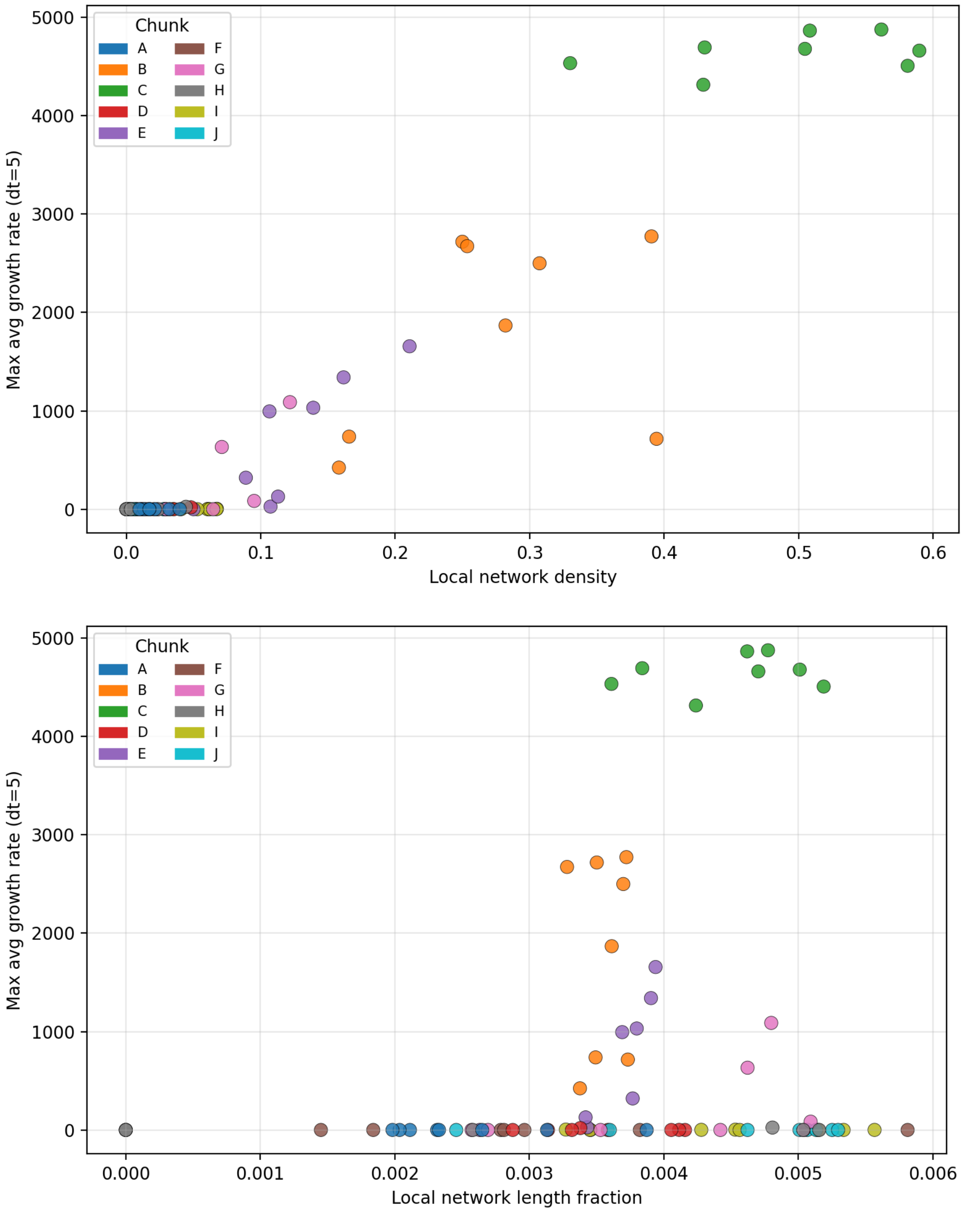

Fig. S2 Maximal tumor growth rate across vascular environments. Shown are the maximal growth rates averaged over 5000 MCS extracted from individual simulations as a function of local vascular properties (density and network length fraction). While qualitatively consistent with the trends observed for final tumor size, growth rates are strongly correlated with final tumor size and therefore do not provide additional independent insights.

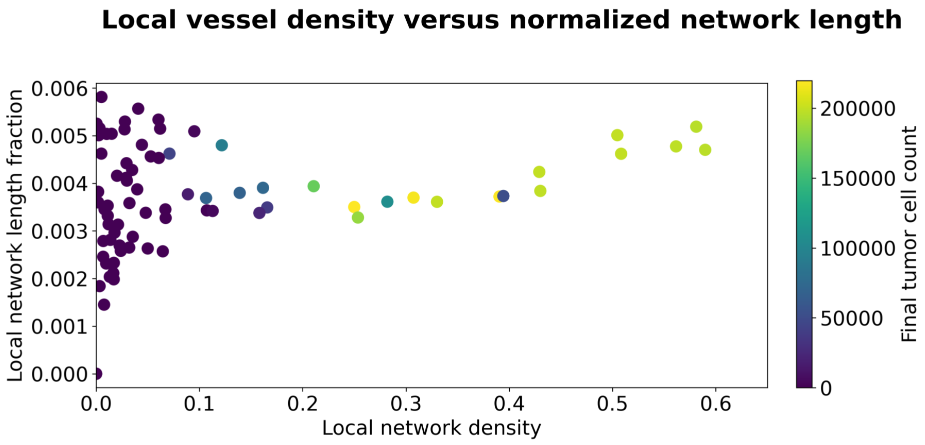

Fig. S3 SI: Mousebrain vessel property comparison. Shown are the local blood vessel density versus the local blood vessel network length for eight simulations of each respective microscopy stack chosen in main manuscript section 2.1.4. In each simulation, the tumor was placed at a different starting position, and hence the local vessel properties differ. The color indicates the final tumor cell count of the respective simulation.

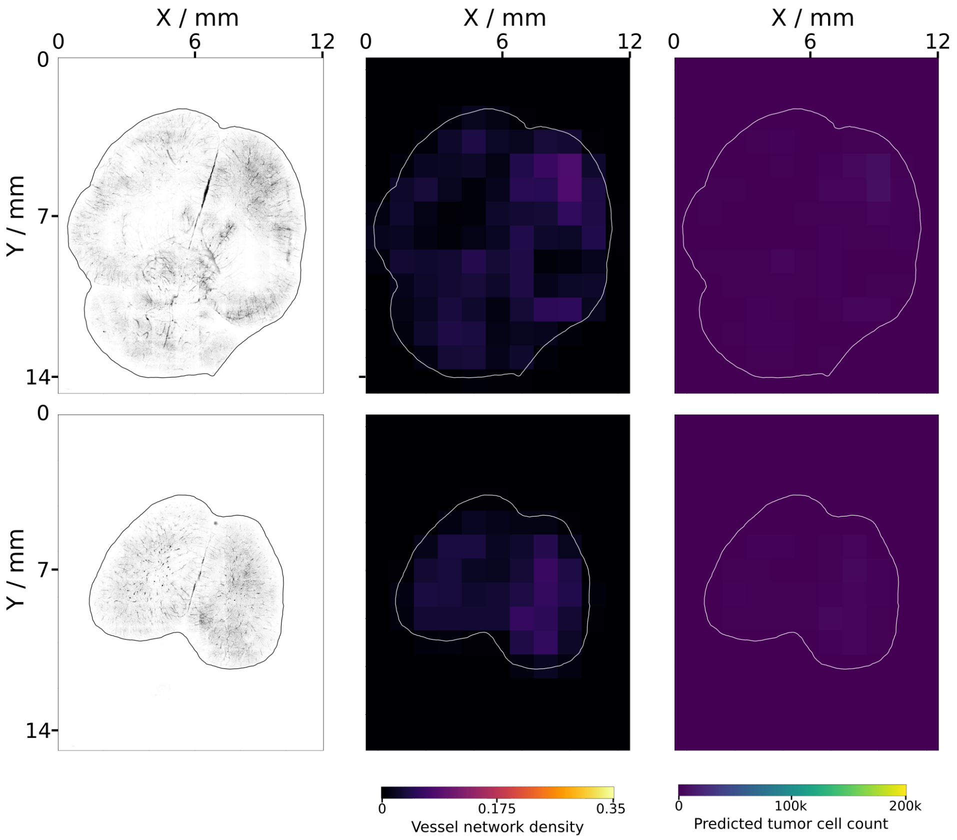

Fig. S4 SI: Mouse brain blood vessel density and predicted tumor growth. Left: Top-down views of the processed and stitched microscopy stacks for the upper two layers of the brain. Middle: heatmaps showing the blood vessel volume fraction for each microscopy stack. Right: heatmaps showing the predicted tumor cell count for each microscopy stack. Predicted values were obtained using main manusript equation 1.

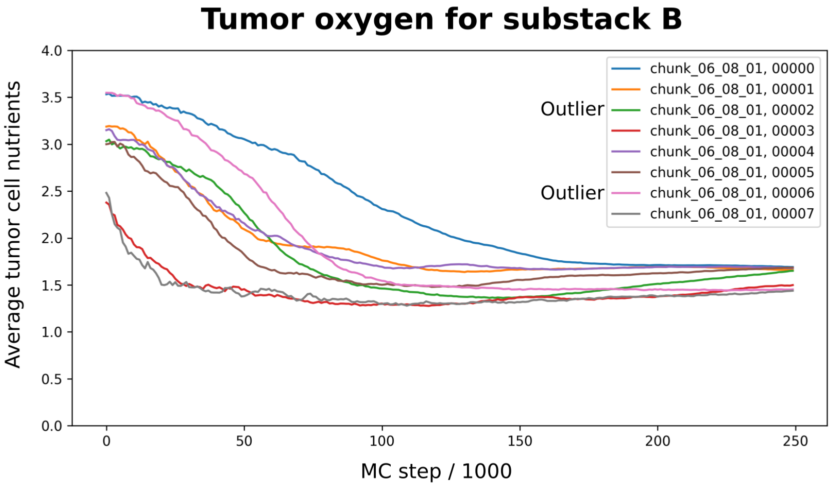

Fig. S5 SI: Tumor cell oxygen content over time for the simulations within the vasculature of substack B (see main manuscript Figure 3). Simulation 2 and 6, which represent the two outliers found in main manuscript Figure 5 b), both start at high nutrient content, with the nutrients of 6 being higher. However, due to a difference in starting position, over the course of the simulation the environment of simulation 2 outside of the sphere in which the local density was calculated is more favourable, and the subsequently the tumor grows faster. Both are hindered by their overall environment and therefore do not reach a final cell count comparable to 0, 1, 4 and 5

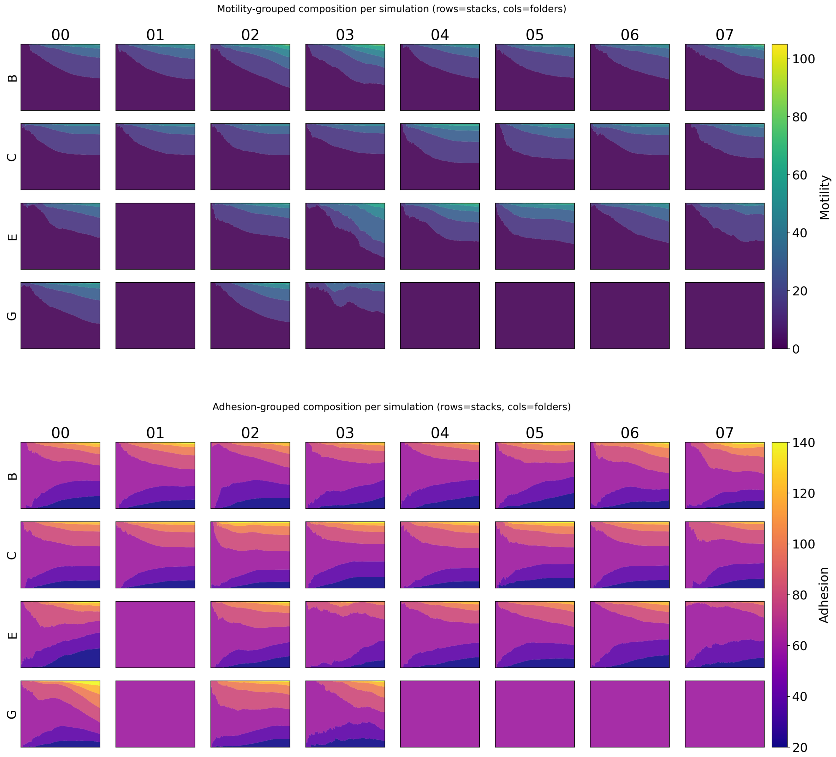

Fig. S6 Tumor composition over time for all fast growing stacks. Grouped by motility (top) and adhesion (bottom).

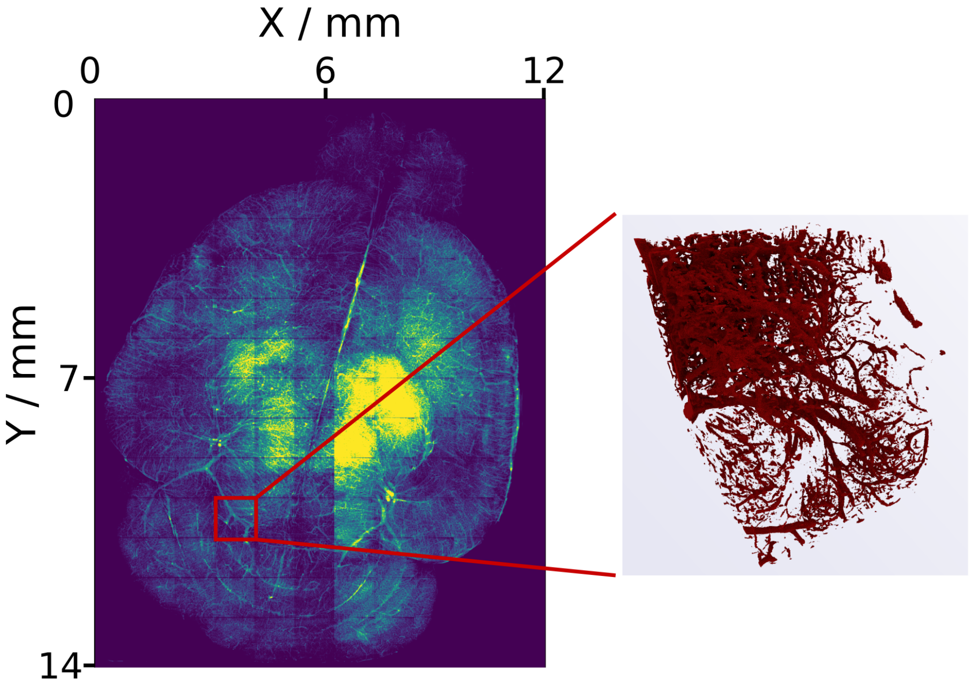

Fig. S7 Mouse brain vasculature data overview. Left: Top-down view of the entire mouse brain. This image consists of 154 concatenated microscopy stacks, indicated by the rectangular artifacts. Each microscopy stack contains 2160 images. Right: 3D-rendering of a single processed microscopy stack. Raw data produced by Di Giovanna et al [1].

Appendix D Mouse brain data candidate stack extraction

All processed mouse brain stacks were compared with respect to the network density ρ and the vessel network length fraction f_l. First, we extracted both parameters for each stack and grouped them using kmeans-clustering, with a cluster count of 10. We chose the stack closest to each centroid for our further studies. The distribution shown in Figure S8 differs slightly from that in Figure 3 of the main manuscript due to improved denoising procedures applied in later analyses, which reduced the influence of noise on the calculation of the network length fraction.

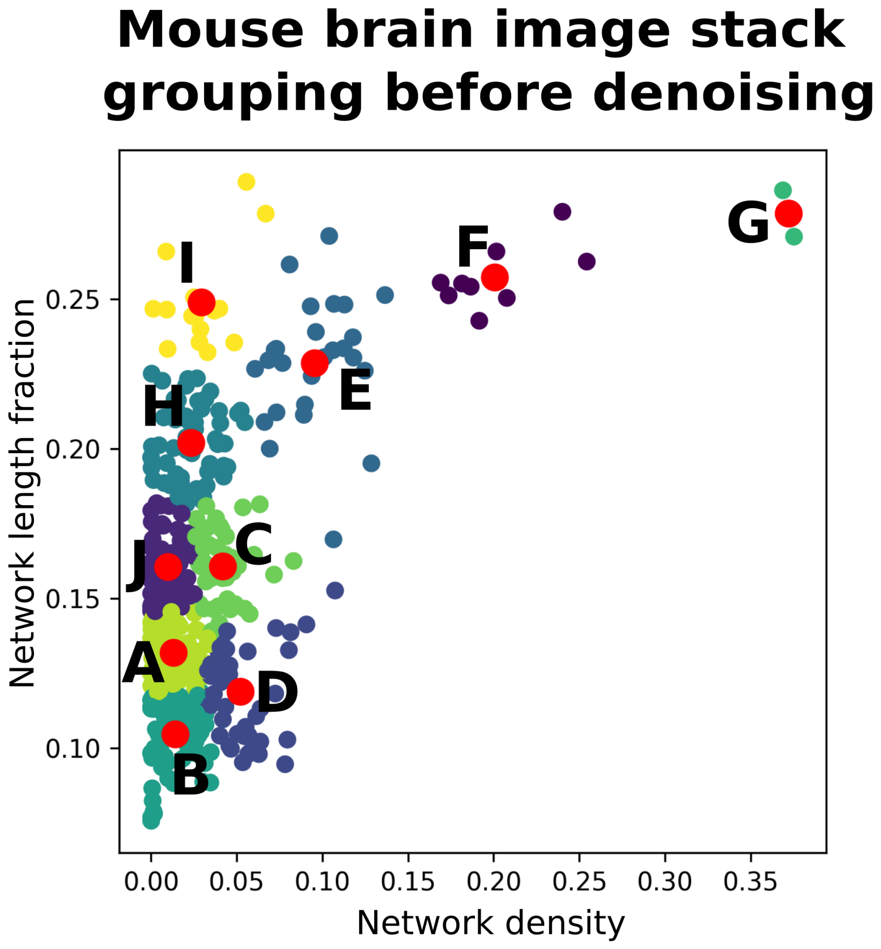

Fig. S8 Extraction of points of interest within processed mouse brain vasculature microscopy stacks. Shown are, for each stack, the network density ρ, and the network length fraction f_l. Each point is color-coded due to cluster-membership after kmeans-clustering, and the red points represent the centroid of each cluster. We chose the stack closest to each centroid for our further studies.

Appendix E Tumor composition analysis: detailed methods

This section explains how we represent tumor-cell phenotypes, how we construct the neutral (no-selection) baseline simulations, and how we infer type-specific selection coefficients from the observed simulations.

Each tumor cell belongs to one of 56 discrete phenotypic types. We label these types by an integer

$$\tau \in S = \{0, 1, \ldots, 55\}.$$

Every type is defined by two underlying quantitative traits:

- Adhesion level, taking values and $\{20, 40, 60, 80, 100, 120, 140\}$
- Motility level, taking values. $\{0, 15, 30, 45, 60, 75, 90, 105\}$

To map the integer type index τ to its adhesion and motility levels, we decompose it as

$$i(\tau) = \left\lfloor \frac{\tau}{8} \right\rfloor \in \{0, \ldots, 6\}, \qquad j(\tau) = \tau \bmod 8 \in \{0, \ldots, 7\},$$

and define

$$A(\tau) = 20\,(i(\tau) + 1), \qquad M(\tau) = 15\,j(\tau).$$

Thus $i(\tau)$ selects one of seven adhesion groups and $j(\tau)$ selects one of eight motility groups.

For each imaging region (called a "stack"), indexed by c, and each replicate simulation indexed by r, we record at every discrete time point t the number of cells of each phenotypic type:

$$\mathbf{n}_{crt} = \big(n_{crt}(\tau)\big)_{\tau \in S}.$$

The total tumor size is

$$N_{crt} = \sum_{\tau} n_{crt}(\tau).$$

The corresponding composition (relative frequencies) is

$$\mathbf{f}_{crt} = \frac{\mathbf{n}_{crt}}{N_{crt}} \in \Delta^{K-1}, \qquad K = |S| = 56.$$

The subscript "crt" therefore indicates: - c: stack, - r: replicate, - t: time point.

Neutral baseline simulations

Let $M \in [0,1]^{K \times K}$ be the mutation matrix describing transitions between types:

$$M_{ij} = \Pr\{\text{offspring type } i \mid \text{parent type } j\}, \qquad \sum_i M_{ij} = 1.$$

If the full mutation matrix includes non-tumor states, we extract the tumor-only submatrix and renormalize each column.

The key distinctions between the two types of simulations are:

- Observed simulations include full tissue mechanics, spatial interactions, adhesion-dependent movement, and selective differences among types.

- Neutral simulations use the same mutation process and the same tumor size trajectory N_{crt}, but no spatial interactions and no selective differences.

To construct the neutral simulation for a given stack and replicate:

1. We take the observed tumor size trajectory $N_{cr0}, N_{cr1}, \ldots$.
2. For each time step, the net growth

$$B_{crt} = \max(0,\ N_{cr,t+1} - N_{crt})$$

   is imposed directly from the observed data.
3. Given the current composition $\mathbf{f}_{crt}$, neutral births are drawn from the mutation process alone:

$$\mathbf{q}^{\text{neu}}_{crt} = M\,\mathbf{f}_{crt}.$$

4. New cells are sampled as

$$\mathbf{b}^{\text{neu}}_{crt} \sim \text{Multinomial}(B_{crt}, \mathbf{q}^{\text{neu}}_{crt}).$$

This produces a neutral trajectory that mirrors the observed growth curve and mutation structure, while removing all effects of selection and spatial tissue dynamics. Thus, differences between observed and neutral compositions reflect selection driven by the full biophysical model.

To infer selection, we model how births occur in the observed simulations. Let the type-specific fitness weights be

$$w_\tau = e^{s_\tau}, \qquad \sum_\tau s_\tau = 0,$$

where the centering constraint ensures identifiability.

Parents are sampled for reproduction at time t according to

$$\mathbf{r}_{crt}(s) = \frac{\text{diag}(\mathbf{w})\,\mathbf{f}_{crt}}{\mathbf{w}^\top \mathbf{f}_{crt}}.$$

Mutation produces offspring-type probabilities

$$\mathbf{q}_{crt}(s) = M\,\mathbf{r}_{crt}(s).$$

Given the net-growth births $\mathbf{b}_{crt}$, the likelihood contribution from time t is

$$\mathbf{b}_{crt} \sim \text{Multinomial}(B_{crt}, \mathbf{q}_{crt}(s)).$$

Summing over all stacks, replicates, and time points with $B_{crt} > 0$ gives the overall log-likelihood $\ell(s)$. Maximizing it yields the estimated selection coefficients $\hat{s}_\tau$.

Neutrality test

Under neutrality $s_\tau = 0$ for all τ, and $\mathbf{q}_{crt} = M\,\mathbf{f}_{crt}$. We compute a likelihood-ratio statistic

$$\Lambda = 2\big(\ell(\hat{s}) - \ell(0)\big),$$

which is asymptotically χ^2_{K-1} distributed.

For interpretability, we aggregate the type-level coefficients across adhesion and motility levels:

$$\overline{s}_a = \frac{1}{|\{\tau : A(\tau) = a\}|} \sum_{\tau : A(\tau)=a} \hat{s}_\tau, \qquad \overline{s}_m = \frac{1}{|\{\tau : M(\tau) = m\}|} \sum_{\tau : M(\tau)=m} \hat{s}_\tau.$$

Implementation notes

Steps with $B_{crt} = 0$ contribute no information about relative fitness and are excluded. A small additive ε inside the log prevents numerical issues. The likelihood naturally weights each time point by its net growth. Pooling across time and replicates stabilizes estimates without additional regularization.

References

[1] Giovanna, D., Paolo, A., Tibo, A., Silvestri, L., Müllenbroich, M.C., Costantini, I., Allegra Mascaro, A.L., Sacconi, L., Frasconi, P., Pavone, F.S.: Whole-brain vasculature reconstruction at the single capillary level. Scientific Reports **8**(1), 12573 (2018)
